# Evaluating Dietary Assessment Methods in Adults with Cystic Fibrosis in the Modulator Era: A Comparison of Food Frequency Questionnaires and Three-Day Food Diaries

**DOI:** 10.3390/nu18183002

**Published:** 2026-09-14

**Authors:** Cian Greaney, Caoimhe Egan, Deanna Kenny, Sarah Tecklenborg, Ciara Howlett, Karen Cronin, Clodagh Landers, Mary Connolly, Derbhla O’Sullivan, Katie Robinson, Audrey Tierney

**Affiliations:** 1 School of Allied Health, University of Limerick, V94 T9PX Limerick, Ireland; 2 Food, Diet and Nutrition Research Group, Health Research Institute, University of Limerick, V94 T9PX Limerick, Ireland; 3 Cystic Fibrosis Ireland, Rathmines, D06 T6H4 Dublin, Ireland; 4 Cork University Hospital, T12 DFK4 Cork, Ireland; 5 St. Vincent’s University Hospital, Dublin 4, D04 T6F4 Dublin, Ireland; 6 University Hospital Galway, H91 YR71 Galway, Ireland; 7 University Hospital Limerick, Dooradoyle, V94 F521 Limerick, Ireland; 8 Aging Research Centre, Health Research Institute, University of Limerick, V94 T9PX Limerick, Ireland; 9 Discipline of Food, Nutrition and Dietetics, La Trobe University, Melbourne, VIC 3086, Australia

**Keywords:** cystic fibrosis, dietary assessment, food frequency questionnaire, food diary, diet quality

## Abstract

**Background/Objectives:** With advances in treatment, nutritional priorities in Cystic Fibrosis (CF) have shifted towards long-term metabolic health. However, uncertainty remains regarding whether commonly used dietary assessment methods provide comparable estimates of dietary intake during this transition. This study aims to compare and evaluate agreement between dietary intakes derived from food frequency questionnaires (FFQs) and three-day food diaries in adults with CF. **Methods:** Cross-sectional study of Irish adults with CF. Semi-quantitative FFQs and three-day food diaries gathered dietary data. **Results:** Forty-four participants were included (variant-specific therapies: 81.8%). No significant differences were observed between methods for energy, carbohydrate, protein, or total fat intake (*p* > 0.05). However, significant differences were identified for several nutrients and food groups. Compared with the three-day food diary, the FFQ reported higher intakes of total sugar, percentage energy from saturated fat, monounsaturated fat, polyunsaturated fat, sodium, zinc, and vitamin E, but lower intakes of fibre and vitamin D (*p* < 0.05). Associations between methods ranged from weak to strong, with the strongest observed for fibre (*r* = 0.701). There were wide limits of agreement for many dietary variables (e.g., energy: −1335.3 to 1242.3 kcal/day), despite comparable group-level estimates. Both methods identified poor adherence to dietary recommendations. **Conclusions:** The FFQ and three-day food diary yielded broadly similar estimates in adults with CF. However, substantial individual-level variability and poor agreement for several nutrients and food groups suggest these methods are not interchangeable for individual dietary assessment. While three-day food diaries may be preferable when detailed assessment of current intake is required, FFQs remain useful for characterising habitual dietary intake in epidemiological and population-based research.

## 1. Introduction

Cystic Fibrosis (CF) is a rare, inherited, life-limiting condition caused by variants in the CF transmembrane conductance regulator (CFTR) gene, following an autosomal recessive mode of inheritance [1]. Dysfunction of the CFTR protein results in dehydrated, viscous secretions affecting multiple organ systems, particularly the lungs, gastrointestinal tract, and pancreas [2]. Consequently, people living with CF (PwCF) commonly experience recurrent pulmonary infections, pancreatic insufficiency, nutrient malabsorption, gastrointestinal complications, and increased energy expenditure associated with chronic inflammation and the increased work of breathing [3].

Historically, malnutrition and underweight represented major clinical concerns in CF [3]. Nutritional management traditionally centred on a high-energy, high-fat dietary approach combined with pancreatic enzyme replacement therapy (PERT) to optimise nutrient absorption and support weight maintenance [4]. This dietary strategy became a cornerstone of CF care due to its association with improved growth, pulmonary function, and survival outcomes [5].

In recent years, the clinical and nutritional landscape of CF has changed substantially following the introduction of highly effective CFTR modulator therapies, also known as variant-specific therapies (VSTs). Although not all PwCF receive these therapies, their widespread availability has fundamentally changed the context of CF care, defining the contemporary ‘modulator era’. Treatments such as elexacaftor/tezacaftor/ivacaftor target the underlying protein defect, contributing to significant improvements in pulmonary outcomes, nutritional status, and quality of life [6,7,8,9]. Registry data now demonstrate median predicted survival exceeding 50 years in several countries [10,11,12].

As survival improves and nutritional status normalises, the nutrition profile of CF is undergoing a significant transition. Historically, overweight and obesity were uncommon in PwCF, but increasing rates are now being reported within the CF population with the most recent Irish registry data outlining that over 40% of adults living with CF are classified as overweight or obese, highlighting changing nutritional challenges within this population [13]. Recent Irish research has also demonstrated suboptimal diet quality among adults living with CF, characterised by high intakes of energy-dense nutrient-poor (EDNP) foods and poor adherence to healthy eating guidelines, despite many individuals failing to meet traditional CF energy targets [14]. These patterns are concerning given the established association between EDNP dietary intake and increased risk of non-communicable diseases in the general population [15].

Importantly, emerging evidence suggests that nutritional transition is occurring at an individual and clinical practice level. Qualitative research has demonstrated that PwCF are already actively modifying eating behaviours in response to weight gain, changing appetite, improved gastrointestinal symptoms, and evolving perceptions of health in the modulator era, while clinicians are increasingly shifting away from the traditional “legacy CF diet” towards dietary approaches that prioritise diet quality and metabolic health [16].

As dietary behaviours and nutritional priorities continue to evolve, accurate assessment of dietary intake becomes increasingly important for both clinical practice and research. Dietary assessment is a key component of CF care [17]. Among the dietary assessment methods used in CF research and clinical practice, food frequency questionnaires (FFQs) and food diaries are among the most commonly employed approaches [18]. FFQs are designed to assess habitual long-term intake by estimating the frequency of food consumption over weeks, months, or years [19], whereas food diaries provide prospective records of foods and beverages consumed over shorter periods, typically several consecutive days [20]. A recent systematic review evaluating dietary intakes in adults living with CF identified substantial heterogeneity in dietary assessment methodologies across studies, including the use of FFQs, food diaries, and 24 h recalls, highlighting ongoing uncertainty regarding the most appropriate approaches for assessing dietary intake in this population [18].

This methodological uncertainty may be particularly important in the modulator era, where eating behaviours and nutritional priorities are evolving rapidly. Most recently, the 2024 ESPEN–ESPGHAN–ECFS guideline marked a significant shift in nutritional priorities for CF, noting that while energy adequacy remains important, dietary advice should also emphasise overall diet quality, cardiometabolic health, and alignment with general healthy eating principles. The guideline no longer promotes a universal high-fat diet and instead recommends individualised macronutrient distribution based on nutritional status, treatment response, and comorbidity risk [21].

As many PwCF are increasingly encouraged to transition away from historic high-energy dietary practices towards eating behaviours more reflective of changing health priorities and evolving clinical guidance, assessment tools designed to capture habitual dietary intake may not fully reflect more recent dietary adaptations. Conversely, three-day food diaries may better capture contemporary intake behaviours and have been reported to show stronger agreement with reference dietary records than FFQs in some validation studies, although both methods have recognised limitations and food diaries may not fully represent usual long-term intake [20]. Consequently, uncertainty remains regarding whether different dietary assessment tools capture comparable representations of dietary intake within this rapidly evolving nutritional environment.

Therefore, this study aims to compare dietary intake data obtained using FFQs and three-day food diaries in adults living with CF and to determine the extent of agreement between these dietary assessment approaches. Nutrient and food group intakes derived from both methods will also be compared against healthy eating guidelines in Ireland to evaluate overall diet quality among PwCF. Establishing whether different dietary assessment tools capture similar or divergent representations of dietary intake is important for informing future nutritional research and clinical assessment strategies in the modulator era.

## 2. Materials and Methods

### 2.1. Study Design and Population

This observational, cross-sectional study adheres to the STROBE guidelines [22]. Quantitative data were gathered using a demographic and self-reported health questionnaire, and three-day food diaries and semiquantitative FFQs collected dietary data (September 2021–March 2023). Study material was offered in digital and paper formats. The Electronic Data Capture system, Castor, Version 1.6 (Ciwit B.V., Amsterdam, The Netherlands) collected and managed questionnaire data. Three-day food diary data were recorded digitally by participants using Libro, a dietary assessment platform within Nutritics Version 5.7 Research Edition (Nutritics Ltd., Dublin, Ireland) [23]. Estimating portion sizes through common household measures and documentation of cooking methods were required within food diaries. Data were exported and analysed through Nutritics, generating comprehensive daily and mean energy, macronutrient, and micronutrient intake values using Nutritics, Ireland (2009 Irish Food Composition Database). Dietary intake was also assessed via a semi-quantitative FFQ adapted from the European Prospective Investigation into Cancer (EPIC) FFQ [24], which has been previously validated in an Irish context [25]. Participants recorded their usual frequency with which they consumed foods and beverages over the previous 12 months using predefined frequency categories. The recorded data were analysed using the FFQ EPIC Tool for Analysis (FETA) tool (University of Cambridge, Cambridge, UK) [26], based on version 6 (CAMB/PQ/6/1205) of the EPIC-Norfolk FFQ. The study protocol [27] was approved by the University of Limerick Institutional Review Board and relevant hospital ethics committees, with all participants providing written informed consent. The full recruitment strategy has been previously published [14].

### 2.2. Assessment of Dietary Intake

Both three-day food diaries and a FFQ were used to evaluate dietary intakes and adherence to dietary guidelines. The methodology used to assess dietary intake from the three-day food diaries has been previously published [14]. For the FFQ, all food and beverage items were assigned specific data-entry codes according to the FETA coding guidelines [26]. Codes were allocated based on the frequency with which participants reported consuming individual food items. Certain items, including milk type and quantity, breakfast cereals, cooking fats, and spreadable fats, required specific coding procedures in line with the FETA guidance. Where foods reported by participants did not have a designated nutrient entry code within the FETA system, the most nutritionally comparable alternative was selected following review and consensus among the study authors.

Following data coding, FFQ data were entered into the FETA software (Windows Version 2.53) [26], which generated detailed reports containing macronutrient and micronutrient intake data. Participants with more than 10 missing FFQ responses were excluded, as nutrient reports could not be generated for these individuals using the FETA software. Oral nutritional supplements (ONS) and micronutrient supplements were not included in the FFQ analysis, as the FETA software does not provide specific coding options for these products. Similarly, ONS and micronutrient supplements were not included in the final three-day food diary dietary intake assessment, allowing examination of micronutrient intake from the diet alone and enabling fair dietary comparisons between the three-day food diary and the FFQ. The dietary intakes produced by both assessment methods were thereafter evaluated by assessing adherence to appropriate CF-specific [21] and general population nutrition guidelines [28,29,30,31].

### 2.3. Statistical Analysis

Statistical analysis was conducted using SPSS^®^ Statistics for Windows, Version 29 (IBM Corp., Released 2022). Macronutrient and micronutrient intakes were quantified as absolute intakes and percentage of total energy intake (%TEI) where appropriate. Descriptive statistics were used to summarise baseline participant characteristics. Normality of data distribution was assessed using the Kolmogorov–Smirnov test, with *p* > 0.05 indicating normal distribution. Data are presented as mean ± standard deviation, median (interquartile range), or frequency (*n*, %) where appropriate. Non-normally distributed variables are indicated within tables.

Differences between nutrient intakes derived from the FFQ and three-day food diary were assessed using paired-samples *t*-tests for normally distributed variables and Wilcoxon signed-rank tests for non-normally distributed variables. Pearson’s Chi-square test was used to assess differences between categorical variables. Where relevant, one-way analysis of variance (ANOVA), Mann–Whitney U tests, and Kruskal–Wallis tests were used to compare continuous variables between groups, with post hoc analyses performed for pairwise comparisons where appropriate.

Agreement between dietary intake estimates obtained from the FFQ and three-day food diary was assessed using Bland–Altman analysis. For each dietary variable, the distribution of the paired differences between methods was assessed prior to the calculation of mean bias and limits of agreement. Pearson’s correlation coefficients (non-parametric: Spearman’s rank correlation) were also calculated to assess the strength and direction of associations between nutrient intakes derived from both dietary assessment methods.

## 3. Results

### 3.1. Demographic and Clinical Characteristics

Table 1 presents the demographic and clinical characteristics of participants who completed both the FFQ and three-day food diary and were eligible for inclusion in the analysis. Participants were excluded if they did not return the food diary following three requests or if the returned diary contained insufficient information for analysis. An additional seven participants who reported taking ONS were excluded, as these products could not be accounted for in the FFQ analysis. A further 17 participants were excluded because the FETA software could not generate nutrient reports for FFQs containing more than 10 missing responses. A total of 44 paired FFQs and three-day food diaries were included.

The mean age of participants was 34.4 ± 8.7 years, and 59.0% were female. The overall mean body mass index (BMI) was 23.9 ± 3.8 kg/m^2^, with male participants demonstrating significantly higher BMI values compared to females (25.3 kg/m^2^ vs. 22.3 kg/m^2^, *p* = 0.001). According to World Health Organisation (WHO) classifications [32], 40.9% of participants were living with overweight or obesity, with significantly higher prevalence observed among males compared to females (61.2% vs. 26.9%, *p* = 0.001).

Pancreatic insufficiency was present in 70.5% of participants, while 18.2% reported CF-related diabetes. Mean predicted percentage forced expiratory volume (FEV_1_%) was 79.3 ± 25.9. The majority (86.4%) of participants reported use of fat-soluble vitamin supplementation (not included in analysis), and 81.8% reported the use of variant-specific therapies.

### 3.2. Comparison of Dietary Intakes and Diet Quality Between the FFQ and Three-Day Food Diary

Energy, macronutrient, micronutrient, and food group intakes derived from the FFQ and three-day food diary are presented in Table 2, alongside paired sample *t*-test, correlation and Bland–Altman agreement analyses. Table 3 presents the proportion of participants meeting dietary recommendations for key nutrients and food groups. Appendix A Table A1 presents recommended nutrient intakes according to CF-specific [21] and general population nutrition guidelines [28,29,30,31].

#### 3.2.1. Energy and Macronutrients

Energy intake was comparable between dietary assessment methods, with median intakes of 2073.1 (880.5) kcal/day from the FFQ and 2109.5 (758.9) kcal/day from the three-day food diary (*p* = 0.641). Similarly, median percentage estimated average requirement (%EAR) did not significantly differ between methods (FFQ: 96.9 (42.3)%; three-day food diary: 104.7 (36.2)%; *p* = 0.699). No significant differences were observed between dietary assessment methods for carbohydrate, protein, or fat intake. Median carbohydrate intake was 218.2 (129.7) g/day using the FFQ and 236.7 (83.4) g/day using the three-day food diary (*p* = 0.927), while median protein intake was 89.7 (43.0) g/day and 96.6 (40.3) g/day, respectively (*p* = 0.333). Median fat intake was also similar between methods (FFQ: 89.9 (39.9) g/day; three-day food diary: 89.3 (42.4) g/day; *p* = 0.606).

#### 3.2.2. Micronutrients

Significant differences between dietary assessment methods were identified for several nutrients. Fibre intake was significantly lower when assessed using the FFQ (14.3 (9.6) g/day) compared with the three-day food diary (19.5 (9.1) g/day; *p* < 0.001). Conversely, the FFQ reported significantly greater intakes of total sugar (FFQ: 96.8 (73.6) g/day; three-day food diary: 92.0 (53.1) g/day; *p* = 0.002), %TEI from total sugar (FFQ: 20.2 (6.9)%; three-day food diary: 16.3 (8.1)%; *p* < 0.001), %TEI from saturated fat (FFQ: 16.4 (4.9)%; three-day food diary: 14.0 (3.8)%; *p* = 0.003), monounsaturated fat (FFQ: 33.4 (13.9) g/day; three-day food diary: 25.5 (22.9) g/day; *p* = 0.006), polyunsaturated fat (FFQ: 11.3 (8.0) g/day; three-day food diary: 10.2 (9.2) g/day; *p* = 0.010), sodium (FFQ: 2829.1 (1462.4) mg/day; three-day food diary: 2568.4 (1069.3) mg/day; *p* = 0.026), zinc (FFQ: 10.7 (4.6) mg/day; three-day food diary: 9.3 (6.7) mg/day; *p* = 0.024), and vitamin E (FFQ: 9.9 (5.8) mg/day; three-day food diary: 8.0 (6.8) mg/day; *p* = 0.001). Vitamin D intake was significantly higher when assessed using the three-day food diary (3.5 (3.9) µg/day) compared with the FFQ (2.1 (1.8) µg/day; *p* = 0.026). No significant differences were observed for calcium, iron, vitamin A, or vitamin C intake between dietary assessment methods.

#### 3.2.3. Food Groups

Significant differences in food group intakes were observed between dietary assessment methods. Compared with the FFQ, the three-day food diary recorded significantly greater intakes of grains (FFQ: 2.3 (0.8) serves/day; three-day food diary: 3.5 (1.8) serves/day; *p* = 0.008), milk, yoghurt and cheese (FFQ: 2.0 (0.8) serves/day; three-day food diary: 2.7 (2.5) serves/day; *p* < 0.001), fats, spreads and oils (FFQ: 1.9 (0.9) serves/day; three-day food diary: 3.3 (2.9) serves/day; *p* < 0.001), and EDNP foods (FFQ: 2.3 (2.0) serves/day; three-day food diary: 5.2 (4.3) serves/day; *p* < 0.001). Conversely, the FFQ reported significantly greater intakes of vegetables, salad and fruit (FFQ: 3.0 (4.3) serves/day; three-day food diary: 2.7 (2.7) serves/day; *p* = 0.029) and protein foods (FFQ: 5.8 (1.2) serves/day; three-day food diary: 3.0 (1.9) serves/day; *p* < 0.001).

#### 3.2.4. Correlation and Bland–Altman Analysis

Correlation analyses demonstrated positive significant associations between nutrient intakes derived from the FFQ and three-day food diary for most nutrients assessed. The strongest positive and significant correlation was observed for fibre intake (*r* = 0.701), followed by %EAR (*r* = 0.612), zinc intake (*r* = 0.583), total sugar intake (*r* = 0.549), and energy intake (*r* = 0.546). Weaker non-significant correlations were observed for percentage energy contribution from protein (*r* = 0.161), percentage fat from monounsaturated fat (*r* = 0.024), and percentage fat from polyunsaturated fat (*r* = 0.242).

**Figure 1:**
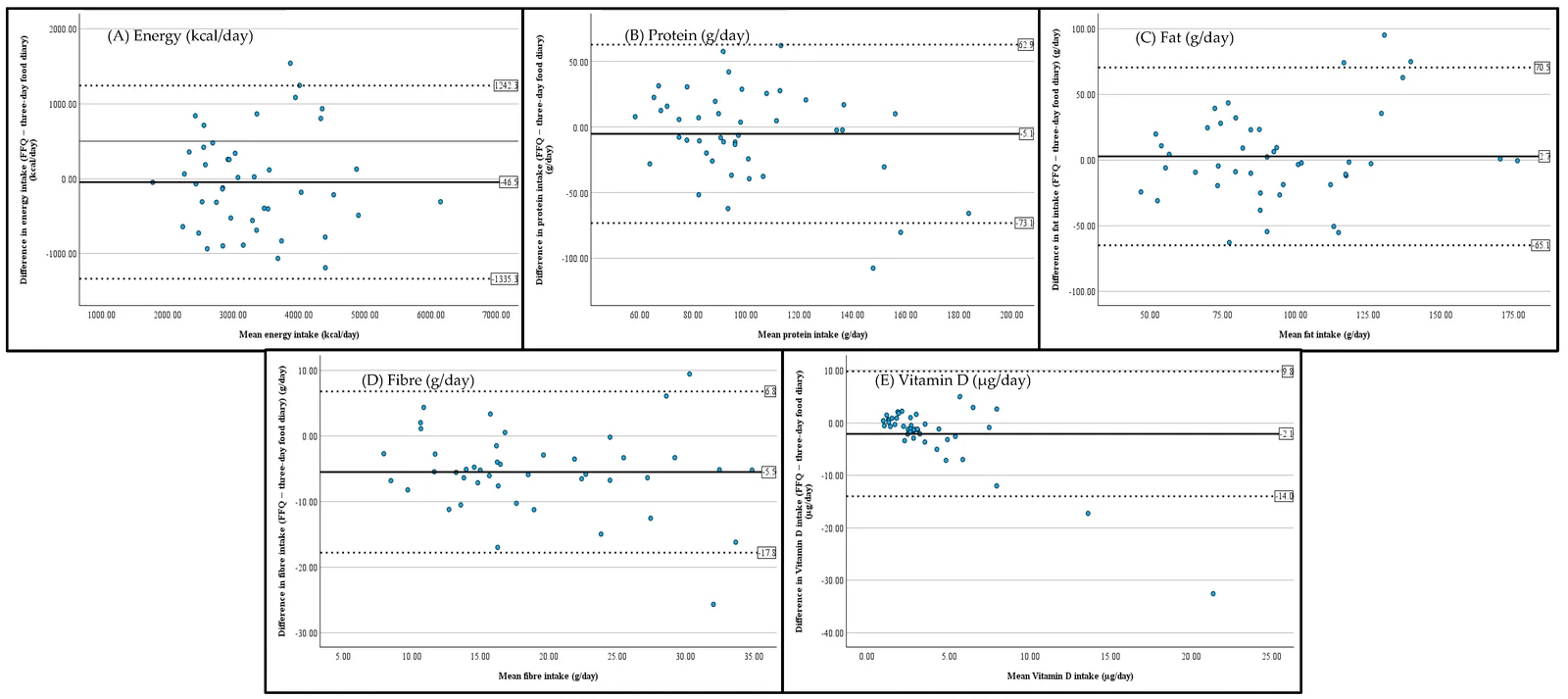
Representative Bland–Altman plots showing agreement between dietary intake estimates derived from the Food Frequency Questionnaire (FFQ) and three-day food diary for (**A**) energy intake, (**B**) protein intake, (**C**) fat intake, (**D**) fibre intake, and (**E**) vitamin D intake. The solid line represents the mean bias, while the dashed lines represent the 95% limits of agreement.

Bland–Altman analysis demonstrated variable agreement between dietary assessment methods across nutrients. The FFQ slightly underestimated total energy intake compared with the three-day food diary, with a mean bias of −46.5 kcal/day and limits of agreement ranging from −1335.3 to 1242.3 kcal/day. Visual inspection of the Bland–Altman plot demonstrated no obvious evidence of proportional bias for energy intake, with differences remaining relatively consistent across the range of mean energy intakes (Figure 1). Underestimation by the FFQ was also observed for protein (−5.1 g/day), fibre (−5.5 g/day), and vitamin D (−2.1 µg/day). Despite the significant difference observed for fibre intake between methods, fibre demonstrated the strongest correlation coefficient (*r* = 0.701), suggesting that the FFQ ranked individuals similarly to the three-day food diary but tended to systematically underestimate absolute fibre intake. In contrast, the FFQ overestimated total sugar intake by 22.3 g/day, sodium intake by 453.7 mg/day, and monounsaturated fat intake by 6.9 g/day compared with the three-day food diary.

Wide limits of agreement were observed for several nutrients, indicating substantial individual-level variability between dietary assessment methods. For example, although mean energy intake differed by only 46.5 kcal/day between methods, the limits of agreement spanned approximately ±1300 kcal/day, indicating that agreement was considerably poorer at the individual level than at the group level. Similar patterns were observed for protein and fat intake. Conversely, fibre and vitamin D demonstrated narrower limits of agreement, although several outlying observations were evident for vitamin D intake.

### 3.3. Comparison of Key Nutrient and Food Group Intakes Derived from the FFQ and Three-Day Food Diary Against Dietary Guidelines

Table 3 demonstrates poor adherence to both CF-specific [21,33] and general healthy eating recommendations [28,30,31] across both dietary assessment methods. Using the FFQ, 63.6% of participants failed to achieve the recommended minimum energy intake requirement of 110% EAR for PwCF, compared with 61.4% using the three-day food diary. Mean %TEI from fat exceeded the recommended range of 20–35% in most participants using both methods, with 81.8% of participants exceeding recommendations according to the FFQ and 68.2% according to the three-day food diary. Similarly, 97.7% and 100.0% of participants exceeded recommendations for %TEI from total sugar using the FFQ and food diary, respectively, while %TEI from saturated fat exceeded recommendations in 88.6% and 84.1% of participants. Fibre intake remained below the recommended intake of 25 g/day for 86.4% of participants using the FFQ and 68.2% using the three-day food diary.

Food group analysis also demonstrated poor adherence to Healthy Ireland National Dietary Guidelines [31]. Most participants failed to achieve recommended intakes for vegetables, salad and fruit using both dietary assessment methods (FFQ: 70.5%; three-day food diary: 82.4%). Similarly, most participants failed to meet grain recommendations when assessed using the FFQ (70.1%), although adherence was better when assessed using the three-day food diary (34.2% below recommendations). Intakes of EDNP foods exceeded recommendations in many participants using both methods, with 86.4% and 94.4% of participants consuming more than one serve per day according to the FFQ and three-day food diary, respectively.

## 4. Discussion

The aim of the present study was to compare dietary intake estimates derived from a FFQ and three-day food diary in Irish adults living with CF and to evaluate the extent of agreement between these commonly used dietary assessment methods. As nutritional priorities shift from a predominant focus on energy adequacy towards diet quality and long-term metabolic health [21], accurate dietary assessment is increasingly important in CF. Overall, the findings demonstrated moderate agreement between methods at a group level, but substantial variability at an individual level. While both methods generated broadly comparable estimates of overall energy and macronutrient intake across the cohort, significant differences were observed for several nutrients and food groups, and Bland–Altman analyses demonstrated wide limits of agreement for many dietary variables. Together, these findings suggest that FFQs and three-day food diaries may provide comparable estimates of dietary intake at a population level, but should not be considered interchangeable for individual dietary assessment in adults living with CF.

### 4.1. Differences Between FFQs and Three-Day Food Diaries

The absence of significant differences in total energy intake, %EAR, carbohydrate intake, protein intake, and fat intake suggests reasonable comparability between methods for assessing overall dietary intake patterns within this cohort. Median energy intake was comparable between methods, and both approaches identified that most participants failed to achieve the minimum recommended energy intake target of 110% EAR for PwCF.

However, despite comparable group-level estimates, Bland–Altman analyses demonstrated substantial variability between methods at an individual level. For example, although the mean bias for energy intake was relatively small (−46.5 kcal/day), limits of agreement ranged from −1335.3 to 1242.3 kcal/day. Similar wide limits of agreement were observed for protein, carbohydrate, sodium, and vitamin A intake. These findings indicate that while both methods may produce similar average estimates across a population, considerable disagreement may exist when assessing individual participants. Therefore, FFQs and three-day food diaries should not be considered interchangeable when precise estimation of individual dietary intake is required. Furthermore, nutrients for which no statistically significant differences were observed should not be interpreted as demonstrating equivalence between methods. Rather, interpretation should consider the combined evidence from paired comparisons, correlation analyses, and Bland–Altman agreement.

There were significant differences observed for several nutrients and food groups. Compared with the three-day food diary, the FFQ reported significantly greater intakes of total sugar, percentage energy from total sugar, percentage energy from saturated fat, monounsaturated fat, polyunsaturated fat, sodium, zinc, and vitamin E, while fibre and vitamin D intakes were significantly lower. Significant differences were also observed for vegetables, salad and fruit, grains, milk, yoghurt and cheese, protein foods, fats, spreads and oils, and EDNP foods. These discrepancies likely reflect a combination of inherent methodological differences between dietary assessment methods and other sources of measurement error. FFQs are designed to assess habitual dietary intake over extended periods [19], whereas food diaries prospectively capture dietary intake over a shorter timeframe and may be more sensitive to recent dietary behaviours and short-term dietary adaptations [20]. However, the limited recording period of food diaries, day-to-day variation in dietary intake, behavioural reactivity during recording, and potential under-reporting of socially undesirable foods may also have contributed to the observed differences between methods, particularly for food group estimates.

This distinction may be particularly relevant within the modulator era. Emerging evidence suggests that many PwCF are modifying dietary behaviours in response to weight gain, improved gastrointestinal symptoms, changing body composition, and evolving nutritional guidance following initiation of VSTs [16,34,35]. While FFQs may capture dietary behaviours that reflect longer-term habitual eating patterns, food diaries may better reflect more recent dietary practices. In a population undergoing nutritional transition, these methodological differences may contribute to discrepancies between dietary assessment methods. However, further research is required to determine the extent to which such differences influence dietary assessment in adults living with CF during the modulator era.

The significantly greater fibre intake observed using the three-day food diary may be relevant in this context. Increasing emphasis is being placed on diet quality and alignment with general healthy eating recommendations [21] in the modulator era. It is therefore possible that higher fibre intakes recorded using food diaries may reflect evolving dietary behaviours that are more readily captured through prospective dietary recording. Nonetheless, it is also plausible that methodological differences between assessment tools may be contributing to these observations.

Similarly, the significantly greater intakes of grains, milk, yoghurt and cheese, fats and oils, and EDNP foods recorded using the three-day food diary may suggest that foods consumed variably or episodically are more likely to be captured through prospective recording than through FFQs [19,20]. Consistent with this interpretation, agreement between methods appeared weaker for several food groups than for many nutrient variables. Weak correlations were observed for protein foods and fats, spreads and oils. Grain intake demonstrated essentially no correlation between methods (*r* = 0.003), indicating that the FFQ and three-day food diary showed no meaningful agreement in ranking individuals according to grain intake. This may reflect the episodic nature of consumption of certain food groups, difficulties in estimating serving sizes, or differences in how foods are categorised within each assessment tool. Collectively, these findings suggest that estimates of food group consumption may be particularly sensitive to the dietary assessment method employed.

Moderate positive correlations were observed between dietary assessment methods for several nutrients, including energy intake, fibre, total sugar, zinc, and %EAR, indicating that participants with relatively higher intakes according to one method generally also reported relatively higher intakes according to the other. However, weaker correlations were observed for percentage energy contributions from protein and monounsaturated fat. Importantly, correlation analyses assess the ability of a dietary assessment method to rank individuals according to intake rather than agreement between methods themselves. Therefore, moderate or even strong correlations should not be interpreted as evidence that two dietary assessment methods are interchangeable. Although several nutrients demonstrated moderate correlations, the wide Bland–Altman limits of agreement observed throughout the present study indicate substantial variability. This was exemplified by fibre intake, which demonstrated the strongest correlation between methods but nevertheless showed systematic differences in estimated intake between the FFQ and three-day food diary, illustrating that good ranking ability does not necessarily equate to agreement in absolute dietary intake estimates. These findings suggest that while FFQs and three-day food diaries may similarly rank individuals according to dietary intake and identify broad dietary patterns within a population, the absolute dietary intake estimates derived from each method frequently differ, limiting their interchangeability for individual dietary assessment.

### 4.2. Adherence to Dietary Recommendations

Poor adherence to both CF-specific [21,33] and general healthy eating recommendations [31] was evident irrespective of dietary assessment method. Most participants exceeded recommendations for saturated fat, total sugar, and EDNP food consumption, while failing to achieve recommended intakes for fibre, fruit and vegetables, and grains. Approximately two-thirds of participants failed to achieve the minimum recommended energy intake target of 110% EAR, while fibre intake remained substantially below recommended levels according to both dietary assessment methods. This observation further highlights fibre as a key nutritional concern within this cohort irrespective of the assessment method used.

These findings are consistent with our previous analysis of dietary intake and diet quality within this cohort using three-day food diary data (*n* = 68), which demonstrated excessive intakes of saturated fat, sugar, and EDNP foods alongside inadequate intakes of fibre, fruit, and vegetables [14]. The present study extends these findings by demonstrating that similar conclusions regarding dietary inadequacies are reached using both FFQs and three-day food diaries despite differences in absolute nutrient and food group estimates between methods.

Importantly, both dietary assessment methods identified broadly similar dietary patterns and nutritional priorities despite differences in absolute nutrient and food group estimates. The consistency of findings across methods supports, but does not prove, that these dietary inadequacies reflect underlying dietary patterns rather than differences arising from the dietary assessment methods used.

These findings remain clinically relevant given the increasing emphasis on diet quality and cardiometabolic health within contemporary CF care [21]. The high prevalence of excessive saturated fat and sugar intake combined with inadequate fibre, fruit, vegetable, and grain consumption observed in the present study highlights a potential mismatch between current dietary behaviours and evolving nutritional recommendations for adults living with CF.

### 4.3. Implications for Practice and Research

The findings of the present study should be considered within the context of the evolving nutritional landscape of CF care in the modulator era. As nutritional priorities extend beyond energy adequacy to include long-term metabolic health and adherence to healthy eating recommendations [21], accurate dietary assessment is increasingly important. Taken together, the findings highlight the importance of selecting dietary assessment methods according to the intended purpose of assessment. Despite substantial variability between methods at an individual level, both FFQs and three-day food diaries identified similar dietary patterns, nutritional priorities, and areas of poor adherence to dietary recommendations. At the population level, both methods produced broadly comparable estimates of overall energy and macronutrient intake. However, greater discrepancies were observed for several micronutrients and food groups, indicating that these outcomes are more sensitive to the dietary assessment method employed.

However, the rapidly evolving nutritional landscape of CF care may influence the choice of dietary assessment method in specific clinical and research contexts. As many PwCF modify dietary behaviours in response to VSTs and evolving nutritional guidance, prospective dietary assessment methods may be better positioned to capture current dietary practices. Furthermore, the ability of three-day food diaries to capture oral nutritional supplement use and provide estimates for nutrients not available through the FFQ used in the present study may represent an additional advantage when a more comprehensive assessment of dietary intake is required. Given the frequent use of ONS in CF and the clinical relevance of micronutrient intake and overall dietary quality [21], these considerations may be particularly important when selecting dietary assessment methods for CF research and clinical practice.

The evolving nutritional landscape of CF care may also necessitate continued innovation in dietary assessment methodologies. Traditional approaches such as FFQs and food diaries remain valuable, but both are reliant on self-report, contributing to participant burden, as well as being susceptible to recall and reporting bias [19,20]. Emerging technologies, including smartphone-based dietary assessment applications, image-assisted dietary recording, wearable devices, and artificial intelligence-assisted dietary analysis, offer opportunities to capture dietary intake in a more objective, real-time, and less burdensome manner [36]. As nutritional management in CF increasingly focuses on diet quality, long-term metabolic health, and individualised care, integration of these technologies into CF research and dietetic practice may help improve the accuracy and clinical utility of dietary assessment.

### 4.4. Strengths and Limitations

The present study has several important strengths. To our knowledge, this is the first study to directly compare dietary intake estimates derived from FFQs and three-day food diaries in adults living with CF within the modulator era. Multiple complementary statistical approaches, including paired comparisons, correlation analyses, and Bland–Altman agreement analyses, were employed to provide a comprehensive evaluation of method performance. Furthermore, assessment of both nutrient intakes and food group consumption enabled evaluation of agreement across a broad range of dietary variables and facilitated examination of adherence to contemporary dietary recommendations.

Several limitations should also be acknowledged. Information on CFTR modulator subtype was not collected, preventing comparisons between specific modulator therapies. The relatively modest sample size may limit generalisability and reduce precision of agreement estimates. The FETA software used to analyse FFQ data [26] did not provide nutrient codes for almond milk or ONS and did not generate nutrient intake values for vitamin K, free sugars, omega-3 fatty acids, or omega-6 fatty acids. As a result, these nutrients could not be compared between dietary assessment methods. Participants with more than 10 missing FFQ responses, as well as those consuming ONS, were excluded from analyses, which may have introduced selection bias and reduced representativeness. As multiple nutrient and food group comparisons were performed without adjustment for multiple testing, the possibility of type I error should be considered when interpreting statistically significant findings, particularly for individual nutrients with *p*-values close to the significance threshold. Accordingly, these findings should be interpreted with appropriate caution and confirmed in larger studies. Furthermore, both dietary assessment methods relied on self-reports and are therefore susceptible to recall bias, social desirability bias, and misreporting. In the absence of an objective reference method (e.g., recovery biomarkers: doubly labelled water for energy, 24 h urinary nitrogen for protein, urinary sodium/potassium), the present study cannot determine which dietary assessment method provides the more accurate estimate of dietary intake [37]. Nevertheless, the use of multiple complementary statistical approaches provides confidence in the overall conclusions regarding agreement between dietary assessment methods.

## 5. Conclusions

In conclusion, significant differences in absolute nutrient and food group estimates, together with wide limits of agreement for several dietary variables, indicate that FFQs and three-day food diaries should not be considered interchangeable when assessing individual dietary intake. Nevertheless, the methods produced broadly comparable estimates of dietary intake at a group level and identified similar patterns of poor adherence to dietary recommendations and suboptimal diet quality among adults living with CF.

While findings suggest that both methods may be useful for identifying broad dietary patterns and nutritional priorities at a population level, greater discrepancies for several micronutrients and food groups indicate that the choice of dietary assessment method is likely to be more important when these outcomes are of primary interest. Three-day food diaries may be preferable when more detailed and comprehensive assessment of dietary intake is required. In addition to demonstrating greater agreement for several dietary variables, three-day food diaries can capture oral nutritional supplement use and provide estimates for nutrients not available through the FFQ used in the present study, several of which are of clinical relevance in CF. Given the rapidly evolving nutritional landscape of CF care in the modulator era, where dietary behaviours may be adapting in response to changing health priorities, treatment advances, and evolving nutritional guidance, consideration should also be given to whether the assessment objective is to capture long-term dietary patterns or more recent dietary behaviours. Careful selection of dietary assessment methods will therefore be important to ensure they are appropriate for the intended clinical or research purpose.

## Figures and Tables

**Table 1:** Demographic and clinical characteristics of Irish adults living with CF who completed an FFQ and a three-day food diary in this cross-sectional study.

	All (*n* = 44)	Male (*n* = 18)	Female (*n* = 26)	*p*-Value
**Age ** **(years) (range 19–68)**	34.4 ± 8.7	38.1 ± 9.3	31.9 ± 7.4	0.026 *
**BMI (kg/m^2^)**	23.9 (3.8)	25.3 (3.4)	22.3 (4.3)	0.001 *
**BMI Classification ** ***n *** **(%) ** ^~§^				0.001 *
Underweight (<18.5)	0 (0.0)	0 (0.0)	0 (0.0)	–
Normal (18.5–24.9)	26 (59.1)	7 (38.8)	19 (73.1)	–
Overweight (25–29.9)	16 (36.4)	10 (55.6)	6 (23.1)	–
Obese (≥30)	2 (4.5)	1 (5.6)	1 (3.8)	–
**FEV_1_%**	79.3 ± 25.9	76.1 ± 29.9	81.5 ± 23.1	0.528
**FEV_1_% Categories ** ***n *** **(%)**				0.514
<40%	4 (9.0)	3 (16.6)	1 (3.8)	–
40–69.99%	10 (22.7)	4 (22.2)	6 (23.1)	–
70–79.99%	7 (15.9)	3 (16.6)	4 (15.4)	–
≥80%	23 (52.3)	8 (44.4)	15 (57.7)	–
**Hospital admission**				
Since last admission (days) ^~^	801 (1296)	893 (1031)	742 (1086)	0.499
Last admission length (days) ^~^	13.5 (9.0)	13.0 (9.0)	13.5 (10.0)	0.337
Admitted in last year *n* (%)	11 (25.0)	3 (16.7)	8 (30.8)	0.128
**Highest Education ** ***n *** **(%)**				0.306
Secondary level	4 (9.1)	1 (5.5)	3 (11.5)	–
Third level ^‡^	26 (59.1)	9 (50)	17 (65.4)	–
Postgraduate level	14 (31.8)	8 (44.4)	6 (23.1)	–
**Income Status ** ***n *** **(%)**				0.038 *
Full income	22 (50.0)	13 (72.2)	9 (34.6)	–
Part-time income	8 (18.2)	1 (5.5)	7 (26.9)	–
No work-based income	14 (31.8)	4 (22.2)	10 (38.5)	–
**Area of Living ** ***n *** **(%)**				0.318
City	16 (36.4)	8 (44.4)	8 (30.8)	–
Town	7 (15.9)	4 (22.2)	3 (11.5)	–
Village	7 (15.9)	3 (16.6)	4 (15.4)	–
Countryside	14 (31.8)	3 (16.6)	11 (42.3)	–
**Living Situation ** ***n *** **(%)**				0.448
Living alone	5 (11.4)	3 (16.6)	2 (7.7)	–
Living with housemates	8 (18.2)	2 (11.1)	6 (23.1)	–
Living with family/relatives	31 (70.5)	13 (72.2)	18 (69.2)	–
**Clinical Characteristics ** ***n *** **(%)**				
Pancreatic insufficiency	31 (70.5)	13 (72.2)	18 (69.2)	0.835
CF-related diabetes	8 (18.2)	3 (16.6)	5 (19.2)	0.833
GORD	10 (22.7)	4 (22.2)	6 (23.1)	0.948
CF-related liver disease	4 (9.1)	4 (22.2)	0 (0.0)	0.011 *
CF-related bone disease	4 (9.1)	1 (5.5)	3 (11.5)	0.485
**Medication/Supplement Use ** ***n *** **(%) ** ^†^				
VSTs	36 (81.8)	13 (72.2)	23 (88.5)	0.170
Antibiotics	20 (45.5)	10 (55.5)	10 (38.5)	0.273
Mucolytics	8 (18.2)	3 (16.6)	5 (19.2)	0.832
Bronchodilators	21 (47.7)	8 (44.4)	13 (50.0)	0.724
Insulin	7 (15.9)	3 (16.6)	4 (15.4)	0.912
Steroids	5 (11.4)	3 (16.6)	2 (7.7)	0.368
Fat-soluble vitamins	38 (86.4)	13 (72.2)	25 (96.2)	0.023 *

Parametric variables are presented as mean ± standard deviation and non-parametric variables as median (interquartile range). *p*-values presented were derived with chi-square tests, independent sample *t*-tests and ANOVA tests (Non-parametric: Mann–Whitney U and Kruskal–Wallis tests). Significance derived from a *p*-value of <0.05. * *p* < 0.05. ^~^ Non-parametric test. ^§^ World Health Organisation cut-off values. ^†^ Chronic medications and supplements used on a regular basis. ^‡^ University, college or vocational education. Abbreviations: BMI, body mass index; FEV_1_%, percentage of predicted force expiratory volume for age, sex, ethnicity, and height; GORD, gastro-oesophageal reflux disease; VSTs, variant-specific therapies.

**Table 2:** Comparison of energy, nutrient, and food group intakes derived from the FFQ and three-day food diary in Irish adults living with CF, including paired *t*-test, correlation, and Bland–Altman agreement analyses.

Nutrient Measure	FFQ	Three-Day Food Diary	*p*-Value	*r*	Mean Bias	Limits of Agreement
Energy (kcal)	2073.1 (880.5)	2109.5 (758.9) ^~^	0.641	0.546 *	−46.5	−1335.3 to 1242.3
*%EAR * ^†^	96.9 (42.3)	104.7 (36.2) ^~^	0.699	0.612 *	−1.9	−64.8 to 61.1
Carbohydrates (g)	218.2 (129.7)	236.7 (83.4) ^~^	0.927	0.530 *	1.1	−157.5 to 159.7
*%TEI Carbohydrates*	43.7 (6.5)	44.2 (7.8) ^~^	0.223	0.276	4.0	−38.3 to 46.3
Protein (g)	89.7 (43.0) ^~^	96.6 (40.3) ^~^	0.333	0.485 *	−5.1	−73.1 to 62.9
*%TEI protein*	18.2 (5.6)	17.8 (4.6) ^~^	0.345	0.161	2.7	−34.7 to 40.1
Fat (g)	89.9 (39.9)	89.3 (42.4) ^~^	0.606	0.508 *	2.7	−65.1 to 70.5
*%TEI Fat*	40.6 (6.3)	37.1 (6.2) ^~^	0.061	0.330 *	1.8	−10.3 to 13.9
Fibre (g)	14.3 (9.6) ^~^	19.5 (9.1) ^~^	<0.001 *	0.701 *	−5.5	−17.8 to 6.8
Total sugar (g)	96.8 (73.6)	92.0 (53.1) ^~^	0.002 *	0.549 *	22.3	−66.6 to 111.2
*%TEI total sugar*	20.2 (6.9)	16.3 (8.1)	<0.001 *	0.493 *	4.4	−4.0 to 26.6
Saturated Fat (g)	37.4 (19.2)	34.1 (19.7)	0.078	0.488 *	4.1	−25.4 to 33.5
*%TEI Saturated Fat*	16.4 (4.9)	14.0 (3.8)	0.003 *	0.311 *	1.9	−5.9 to 9.6
Monounsaturated fat (g)	33.4 (13.9)	25.5 (22.9) ^~^	0.006 *	0.362 *	6.9	−23.9 to 37.6
*%Fat monounsaturated*	36.2 (3.7)	29.9 (13.2)	<0.001 *	0.024	7.2	−10.6 to 25.0
Polyunsaturated fat (g)	11.3 (8.0)	10.2 (9.2) ^~^	0.010 *	0.407 *	3.0	−11.3 to 17.3
*%Fat polyunsaturated*	13.4 (5.2)	11.1 (6.2) ^~^	0.001 *	0.242	3.1	−7.5 to 13.7
Sodium (mg)	2829.1 (1462.4) ^~^	2568.4 (1069.3) ^~^	0.026 *	0.463 *	453.7	−2097.0 to 3004.4
Calcium (mg)	1003.5 (629.4)	946.0 (699.8)	0.089	0.444 *	126.0	−816.6 to 1068.6
Iron (mg)	10.7 (4.6) ^~^	8.6 (7.3) ^~^	0.154	0.451 *	1.0	−7.9 to 9.9
Zinc (mg)	10.7 (4.6)	9.3 (6.7) ^~^	0.024 *	0.583 *	1.3	−6.0 to 8.7
Vitamin A (ret. eq.) (µg)	962.1 (720.3)	937.6 (1052.4) ^~^	0.084	0.518 *	184.0	−1165.5 to 1533.5
Vitamin D (µg)	2.1 (1.8) ^~^	3.5 (3.9) ^~^	0.026 *	0.322 *	−2.1	−14.0 to 9.8
Vitamin E (mg)	9.9 (5.8)	8.0 (6.8) ^~^	0.001 *	0.343 *	3.6	−9.3 to 16.5
Vitamin C (mg)	77.1 (65.7) ^~^	71.0 (79.9) ^~^	0.138	0.422 *	13.2	−100.2 to 126.6
**Food Serves (serves/day)**						
Vegetables, salad & fruit	3.0 (4.3) ^~^	2.7 (2.7) ^~^	0.029 *	0.629 *	0.8	−3.9 to 5.6
Grains ^‡^	2.3 (0.8) ^~^	3.5 (1.8)	0.008 *	0.003	−1.0	−5.7 to 3.7
Milk, yoghurt & cheese	2.0 (0.8)	2.7 (2.5) ^~^	<0.001 *	0.439 *	−1.2	−4.4 to 2.1
Protein foods ^§^	5.8 (1.2)	3.0 (1.9)	<0.001 *	0.192	2.4	−1.6 to 6.3
Fats, spreads & oils	1.9 (0.9) ^~^	3.3 (2.9)	<0.001 *	−0.149	−1.8	−6.8 to 3.3
EDNP foods ^||^	2.3 (2.0)	5.2 (4.3) ^~^	<0.001 *	0.399 *	−3.4	−10.9 to 4.1

All values in the FFQ and three-day food diary columns were calculated and are reported as median (interquartile range). Variables marked with “^~^” were non-parametric and analysed using non-parametric statistical tests. *p*-values were derived from paired sample *t*-tests (Non-parametric: Wilcoxon signed-rank test). *r* Pearson’s correlation value (Non-parametric: Spearman’s rank correlation). Mean bias and limit of agreement values presented are from the Bland–Altman analysis. Significance derived from a *p*-value < 0.05. * *p* < 0.05. ^†^ Derived from the average energy intake recommendations (female: 1800–2000 kcal/day, male: 2000–2500 kcal/day) provided by Healthy Ireland. ^‡^ Wholemeal cereals and breads, potatoes, pasta, and rice. ^§^ Meat, poultry, fish, eggs, beans and nuts. ^||^ Foods and drinks high in fat, sugar and salt. Abbreviations: FFQ, Food Frequency Questionnaire, %EAR, percentage estimated average requirement; %TEI, percentage of total energy intake from; ret. eq., retinol equivalent; EDNP, Energy-Dense Nutrient Poor.

**Table 3:** Percentage of Irish adults living with CF achieving guideline intakes in this cross-sectional study.

Nutrient Measure	Recommended Intake	Below Recommendation (FFQ)	Below Recommendation (Three-Day Food Diary)	Within Recommendation (FFQ)	Within Recommendation (Three-Day Food Diary)	Above Recommendation (FFQ)	Above Recommendation (Three-Day Food Diary)
%EAR ^†^	110–200 ^a^	63.6	61.4	34.1	36.4	2.3	2.3
%TEI carbohydrates	45–60	59.1	59.1	36.4	40.9	4.5	0.0
%TEI protein	15–20 ^b^	20.5	15.9	50.0	52.3	29.5	31.8
%TEI fat	20–35	0.0	0.0	18.2	31.8	81.8	68.2
Fibre (g)	25	86.4	68.2	13.7	34.1	–	–
%TEI total sugar	<10 ^c^	–	–	2.3	0.0	97.7	100.0
%TEI saturated fat	<10 ^c^	–	–	11.4	15.9	88.6	84.1
**Food ** **Serves (serves/day) ^d^**							
Vegetables, salad & fruit	5–7	70.5	82.4	11.4	10.6	18.2	7.0
Grains ^‡^	3–5 ^e^	70.1	34.2	26.3	49.1	3.6	16.7
Milk, yoghurt & cheese	3	81.8	60.0	18.2	13.7	0.0	25.8
Protein foods ^§^	2	27.3	12.1	31.8	21.6	40.9	66.3
Fats, spreads and oils	Very small amounts	–	–	36.4	19.4	63.6	80.6
EDNP foods ^||^	Not every day	–	–	13.6	5.6	86.4	94.4

Data are presented as percentages. Guideline intakes are EFSA general population dietary reference values [28] unless stated otherwise. ^†^ Derived from the average energy intake recommendations (female: 1800–2000 kcal/day, male: 2000–2500 kcal/day) provided by Healthy Ireland [31]. ^a^ Derived from ESPEN-ESPGHAN-ECFS CF-specific nutrition guidelines [21]. ^b^ Derived from Academy of Nutrition and Dietetics CF-specific nutrition guidelines [33]. ^c^ Derived from the Irish Heart Foundations nutrition guidelines for heart health [30]. ^d^ Derived from Healthy Ireland guidelines [31]. The agreement thresholds described below represent author-defined operational definitions developed for the purposes of this study. For food groups with single recommended intake values, participants were classified as meeting recommendations if intake was within ±0.5 serves/day of the recommended value. For qualitative recommendations, operational cut-offs were applied for analytical purposes, whereby fats, spreads and oils were classified as meeting recommendations if intake was <1.5 serves/day and EDNP foods were classified as meeting recommendations if intake was <1 serve/day. ^e^ Up to 7 serves per day for men 19–50 years old. ^‡^ Wholemeal cereals and breads, potatoes, pasta, and rice. ^§^ Meat, poultry, fish, eggs, beans and nuts. ^||^ Foods and drinks high in fat, sugar and salt. Abbreviations: FFQ, Food Frequency Questionnaire, %EAR, percentage estimated average requirement; %TEI, percentage of total energy intake from; EDNP, Energy-Dense Nutrient Poor; EFSA, European Food Safety Authority.

## Data Availability

The raw data supporting the conclusions of this article will be made available by the authors on request.

## Abbreviations

CF	Cystic Fibrosis
CFTR	Cystic Fibrosis transmembrane conductance regulator
PwCF	People living with Cystic Fibrosis
PERT	Pancreatic enzyme replacement therapy
EDNP	Energy-dense nutrient-poor
FFQ	Food frequency questionnaire
EPIC	European Prospective Investigation into Cancer
FETA	FFQ EPIC Tool for Analysis
ONS	Oral nutritional supplements
%TEI	Percentage of total energy intake
ANOVA	One-way analysis of variance
BMI	Body mass index
WHO	World Health Organisation
FEV_1_%	Predicted Percentage Forced Expiratory Volume
%EAR	Percentage estimated average requirement

## Appendix A

**Table A1:** Recommended Nutrient Intake and Food Serves for Males and Females.

Nutrient Measure	Recommended Intakes		
	All	Male	Female
Energy (kcal)	–	2200–5000 ^a^	1980–4000 ^a^
*%EAR*	110–200 ^a^	–	–
*%TEI carbohydrates*	45–60	–	–
*%TEI protein*	15–20 ^b^	–	–
*%TEI Fat*	20–35	–	–
Fibre (g)	25	–	–
Total sugar (g)	As low as possible	–	–
*%TEI Total sugar*	<10 ^c^	–	–
Saturated Fat (g)	As low as possible	–	–
*%TEI Saturated Fat*	<10 ^c^	–	–
Sodium (mg)	–	2000 ^d^	–
Calcium (mg)	18–24 years: 1000; >25 years: 950	–	–
Iron (mg)	–	11	16
Zinc (mg)	–	9.4–16.3	7.5–12.5
Vitamin A (ret. eq.) (µg)	–	750 ^e^	650 ^e^
Vitamin D (µg)	15 ^e^	–	–
Vitamin E (mg)	–	13 ^e^	11 ^e^
Vitamin K_1_ (µg)	70 ^e^	–	–
Vitamin C (mg)	–	110 ^e^	95 ^e^
**Food Serves (serves/day) ^f^**			
Vegetables, salad & fruit	5–7	–	–
Grains ^‡^	3–5 ^g^	–	–
Milk, yoghurt, & cheese	3	–	–
Protein foods ^§^	2	–	–
Fats, spreads, & oils	Very small amounts	–	–
EDNP foods ^||^	Not everyday	–	–

^a^ Derived from ESPEN-ESPGHAN-ECFS CF-specific guidelines [21]. ^b^ Derived from Academy of Nutrition and Dietetics CF-specific nutrition guidelines [33]. ^c^ Derived from the Irish Heart Foundation’s (IHF) nutrition guidelines for heart health (IHF, 2007). ^d^ CF-specific nutrition guidelines recommend supplementing in stress situations when excessive sweating is expected (i.e., fever, exercise/sports, hot weather) [33]. ^e^ CF-specific nutrition guidelines recommend to always take a supplement on top of recommended intakes [33]. ^f^ Derived from Healthy Ireland guidelines [31]. ^g^ Up to 7 serves per day for men 19–50 years old [31]. Abbreviations: EDNP, Energy-Dense Nutrient Poor. ^‡^ Wholemeal cereals and breads, potatoes, pasta, and rice. ^§^ Meat, poultry, fish, eggs, beans and nuts. ^||^ Foods and drinks high in fat, sugar and salt.
